# Extending the R Library *PROPER* to Enable Power Calculations for Isoform-Level Analysis with *EBSeq*

**DOI:** 10.3389/fgene.2016.00225

**Published:** 2017-01-10

**Authors:** Amadou Gaye

**Affiliations:** Metabolic, Cardiovascular and Inflammatory Disease Genomics Branch, National Human Genome Research InstituteBethesda, MD, USA

**Keywords:** mRNA, isoforms, statistical power, differential expression analysis, transcription, genetic

## Abstract

RNA-Sequencing (RNA-Seq) has become a routine technology for investigating gene expression differences in comparative transcriptomic studies. Differential expression (DE) analysis of the isoforms of genes is just emerging now that expression (read counts) can be estimated with higher accuracy at the isoform level. Estimating the statistical power that can be achieved with a specific number of repeats is a key step in RNA-Seq analysis. The R library proper was developed to provide realistic empirical power analysis. However, proper uses differential expression methods more suited for power calculation of gene-level expression data. We propose extensions to this tool that would allow for power analysis which takes into account the specificities of isoforms expression. This was achieved by enabling the use of *EBSeq*, a DE approach well-tailored for isoform-level expression, as an additional analysis method within *PROPER*. The new extensions and exemplar code for their usage are freely available online at: https://github.com/agaye/proper_extension

## Introduction

PROspective Power Evaluation (*PROPER*) (Wu et al., 2015) is an R library developed for power calculations in RNA-seq differential expression (DE) analysis. It allows one to determine the probability of finding a true variation in expression between two groups (e.g., cases and controls). In addition to the usual parameters included in power estimation such as sample size, effect size, and within-group variance, *PROPER* takes into account other keys characteristics of count data that influence power including the distribution of the mean expression level, the sequencing depth and the threshold for filtering out molecules. By not treating these critical factors as fixed input, the tool avoids strong assumptions such as exchangeability between genes and hence provides more realistic statistical power estimations (Wu et al., 2015) than other methods and closed-form solutions. *PROPER* uses existing DE analysis methods including *edgeR* (Robinson et al., 2010), *DESeq* (Anders and Huber, 2010; Love et al., 2014), and *DSS* (Wu et al., 2013). However, these methods are better suited for gene-level (the summed expression of the distinct isoforms of a transcript) DE analysis because they were not designed to accommodate the differential uncertainty in isoform expression estimation (Leng et al., 2013). A useful extension to *PROPER* would be to include in its machinery a tool such as *EBSeq* that takes into account specificities of isoform-level expression data. *EBSeq* uses an empirical Bayesian approach to model a number of features observed in RNA-seq. This tool is more suited for isoform level inference because it accommodates isoform expression estimation uncertainty by modeling the differential variability observed in distinct groups of isoforms (Leng et al., 2013) and takes into account colinearity between isoforms originating from the same transcript. In *EBSeq*, a posterior probability of being differentially expressed (PPDE) is computed as measure of statistical significance.

## Materials and methods

We amended two functions of *PROPER* (runSims and powerPlot) and wrote two additional functions (run.EBSeq and plotFDR) to enable power calculations that require *EBSeq* and to add new functionality.

### runSims

This is the simulation function of the package *PROPER*; it carries out simulations for a specified number of runs and for a given sample size (two groups of equal size). At each run, a dataset which has the characteristics of the data being investigated is generated and analyzed, i.e., transcripts differentially expressed between the two groups are detected, using one of the differential expression (DE) analysis methods currently available in *PROPER* (*edgeR, DSS* and *DEseq*). After each run the names of the transcripts differentially expressed are returned along with their *p*-value, log fold change and FDR.

The extensions we are proposing consist of changing the argument/parameter that specifies the DE method to add *EBSeq* to the list of methods and altering the core of the function to enable DE analysis using *EBSeq*. Since for isoform analysis, unlike gene-level analysis, isoform names are required in addition to the gene name, we inserted a check to ensure this requirement is met to prevent the function from crashing if input tables similar to those for *edgeR, DEseq*, and *DSS* are provided while *EBSeq* is specified as DE analysis method.

In the implementation of *EBSeq* we enabled within runSims, transcripts significantly differentially expressed are those with PPDE ≥ (1–alpha) where alpha (type I error rate) is set to 0.05; a value that can be changed in the code we provided. In this setting significance is based on alpha so that all transcripts with FDR ≤ alpha are considered significantly differentially expressed and FDR is similar to the idea of bonferroni correction. However it is important to note that *EBSeq* does not require extra multiple test adjustment since it uses a single large model to test all transcripts simultaneously rather than a model testing one gene at a time (Leng et al., 2013). Alpha is also called target FDR which in the setting we described is based on a hard threshold. *EBSeq* offers the possibility to compute a soft threshold via the function crit_fun described in its R manual.

### run.EBSeq

This is the function we wrote to carry out *EBSeq* isoform analysis within *runSims*. It is analogous to the other three functions that can be called to run DE analysis using *egdeR, DEseq*, and *DSS*. The function analyzes the simulated isoform expression data and outputs a matrix with all the transcript, *p*-values, log fold changes, FDR values, and other statistics. This information is then subsequently passed on respectively to comparePower, powerPlot and plotFDR to evaluate power and generate graphs. comparePower is central to *PROPER* as it is the function that computes power by evaluating the success rate over the multiple simulation runs. Success rate is determined by comparing the *p*-values to the specified type I error rate (alpha) to reject to null hypothesis. More details about this function are available from *PROPER* tutorial document.

### powerplot and plotFDR

Our changes to the function powerPlot consisted mainly in amendments to allow for the plotting of just one curve instead of multiple curves (one per sample size considered) and minor additions related to the visualization of the curves. The function plotFDR is similar to powerPlot save it plots FDR curves. There is a function of the same name in *PROPER* and hopefully, the code we generated can be integrated into that function to enable the additional features we introduced.

## Conclusions

We extended the package *PROPER* to allow for transcript isoform analysis and have a more comprehensive tool. It is important to note that for this work we made sure we followed the strategy and nomenclature of the authors of *PROPER* as to not disrupt their excellent work and to make potential future integration of the new functions into their R library easy; and this is the very reason why we kept our code basic as more complexity for increased flexibility could render future integrative more cumbersome. However, until that integration takes place the current functions can be used as standalone scripts that call elements required from *PROPER* and *EBSeq* to carry out statistical power analysis for isoform-level RNA-seq expression data. We also provide a succinct tutorial as a guide for using the new functions. The code in the guide on section guide to using the extensions of this manuscript and the rest of the material (scripts and functions) are available from GitHub at: https://github.com/agaye/PROPER_Extension

## Guide to using the extensions

### Preamble

The aim of this short guide is to demonstrate the use of the new extensions to compute statistical power for isoform expression data using the *EBSeq* approach. An excellent *PROPER* tutorial is available online at: https://www.bioconductor.org/packages/devel/bioc/vignettes/PROPER/inst/doc/PROPER.pdf

This documentation along with the R manual of the *PROPER* package provides a detailed explanation of the parameters we set in the code on this document. Therefore, the arguments required by *PROPER* functions used in this tutorial will not be explained with great details. Similarly we refer the user to *EBSeq* documentation (https://www.rdocumentation.org/packages/EBSeq/versions/1.12.0) for a deeper understanding of that method.

The data used in this guide (see Supplementary Material Datasheet 1) and the scenario investigated are just meant for demonstration and are by no means biologically meaningful. They are only meant to help understand the formats of the input data and the steps to evaluate statistical power and visualize the results.

It is important to note that a real world analysis typically includes more than 10,000 genes and a *PROPER* analysis of such large data can be computationally slow. For example an analysis of 15,000 genes undertaken with 50 simulation runs on a machine with 8GB memory took 2 hours to complete. This is because, in addition to the iterative process of *PROPER*, each model fit in *EBSeq* takes 2 or more iterations to reach convergence. The high computational cost represents a limitation; but we believe this weakness is worth the more realistic results achieved through this approach.

### Input data

The data simulated and analyzed is a matrix where the first 2 columns of the table hold isoforms and transcripts IDs. The process is stopped and a message printed on screen if the information in these 2 columns is missing while the user has specified *EBSeq* as DE analysis method. For the extensions presented in the manuscript to run, the names of the first two columns must be “transcript_id” and “gene_id” and the IDs in the first column must be unique; here we used UCSC IDs but other names such as RefSeq IDs are also valid. The rest of the columns holds sample IDs. As shown below, rows store isoform expression values for the samples in the columns.

The toy data used in this demonstration (Table 1) consist of the expression of 208 isoforms across 8 samples (2 groups: 4 cases and 4 controls). The first 4 samples (ID1 to ID4) represent to control group whilst ID5 to ID10 are the case group. *PROPER* requires an equal size for the 2 groups (e.g., cases and controls).

**Table 1:** **Six of the 8 columns and first 5 rows (5 isoforms of 2 transcripts) of the toy data**.

**Transcript_id**	**Gene_id**	**ID1**	**ID2**	**ID3**	**ID4**	**ID5**	**ID6**
uc003ceg.2	AZI2	42	35	30	13	29	24
uc011axd.1	AZI2	31	72	23	29	37	49
uc003ceb.3	AZI2	1085	1190	843	707	806	948
uc003yky.3	AZIN1	3622	4095	2857	2750	2866	3458
uc003ykx.3	AZIN1	2043	1943	1500	981	1206	1569

Along with this table we provide 3 variables (AveLogCPM, logDispersion, libCount) which represent respectively the log average Count Per Million [average expression of each isoform, CPM = count/sum (counts) x 1 million] log taqwise (genewise) dispersion (dispersion value for each isoform) and the depth of the data (the total count for each sample). This information gives the characteristics of “real” data (actually some hypothetical data) we are analyzing. As already mentioned these parameters are explained with ample details in the *PROPER* tutorial and Manual. For this tutorial we obtained the characteristics of our data from a pilot analysis but as explained by Wu et al. one may also set these parameters based on literature or other sources.

### Power analysis

In *PROPER*, power analysis is carried out in 3 steps: First the simulation scenario is set up then the simulated data are generated with the characteristics of the real data and finally the power is evaluated.

Let us begin by loading the R libraries and functions required. We load the package *edgeR* because we use the Trimmed mean of M-values normalization (TMM) method embedded in that library to normalize the data in the *EBSeq* analysis step.


  > library(PROPER)
  > library(EBSeq)
  > library(edgeR)
  > source("run.EBSeq.R")
  > source("runSims_ag.R")
  > source("plotPower_ag.R")
  > source("plotFDR_ag.R")


Now we load the characteristics of our “real” data and the estimated log fold change of the differentially expressed isoforms which represent a proportion (propDE) of 6% of all the isoforms.

In a real world power analysis it is probably better to obtain these characteristics and a rough estimate of the number of differentially transcripts and their fold change difference from the real data by running a preliminary DE analysis. In such case the analyst can:

1. Estimate dispersion for each transcript (“genewise” dispersion); this is possible by using for example the empirical Bayes strategy (McCarthy et al., 2012) implemented in *edgeR*. Dispersion outliers should not be considered in the results or should be interpreted with caution because outlying dispersion can indicate low quality.
2. Compute the average expression of each transcript by fitting a binomial generalized log-linear model to the expression data (read counts); in such model the dispersion parameter takes the dispersion values calculated as explained in (1). This is possible using the implementation, in *edgeR*, of generalized linear model, GLM (McCarthy et al., 2012).
3. Obtain an estimate of the number of DE transcripts and their log fold change difference from the results of the GLM fit in *edgeR* or from a preliminary DE analysis in *EBSeq*.


  > load("toy_data.RData")
  > load("AveLogCPM.RData")
  > load("logDispersion.RData")
  > load("libCount.RData")
  > load("logFC.RData")
  > propDE  <- 0.06
 


Set the simulation i.e. generate an object that stores the parameters of the simulations.


  > mm  <- RNAseq.SimOptions.2grp(
          Ngenes = dim(toy_expr)[1],
          lBaselineExpr = AveLogCPM,
          seqDepth = libCount,
          lOD = logDispersion,
          p.DE=propDE, lfc=logFC,
          sim.seed=123)


We now can run the simulation, generating n datasets, where n is the number of simulation runs, and analyzing each dataset. We set the number of runs to 20 but in real world example one should select at least 50 runs and the larger the number of runs the more reliable the results are. The argument “Nreps” represents the number of case-control pairs (4 in the toy data). The function returns *p*-values and FDR values for each simulation run.


  runSimsResults  <- runSims_ag(Nreps=4,
               sim.opts=mm,
               DEmethod="EBSeq",
               isoforms=toy_data[,c(1,2)],
               nsims=20)


Finally power is evaluated across all the runs and the results graphically displayed. The arguments and output of the below function are described in details in the R manual for *PROPER*; briefly: The function returns overall and marginal values for power, type I error rate and FDR. In this example we used only the arguments relevant for the isoform analysis process ran by calling *run.EBSeq* from within this function; DE significance is based on FDR and the cut-off for significance is 0.05. We assess power for each expression strata (stratify.by = “expr”) and all the expression strata we specified (strata) are considered (strata.filtered = 0).


  powers  <- comparePower(
       runSimsResults,
       alpha.type = "fdr",
       alpha.nominal = 0.05,
       stratify.by="expr",
       filter.by="none",
       strata=c(0,5,10,2^(1:6)*10,Inf),
       strata.filtered=0)


**Figure 1:**
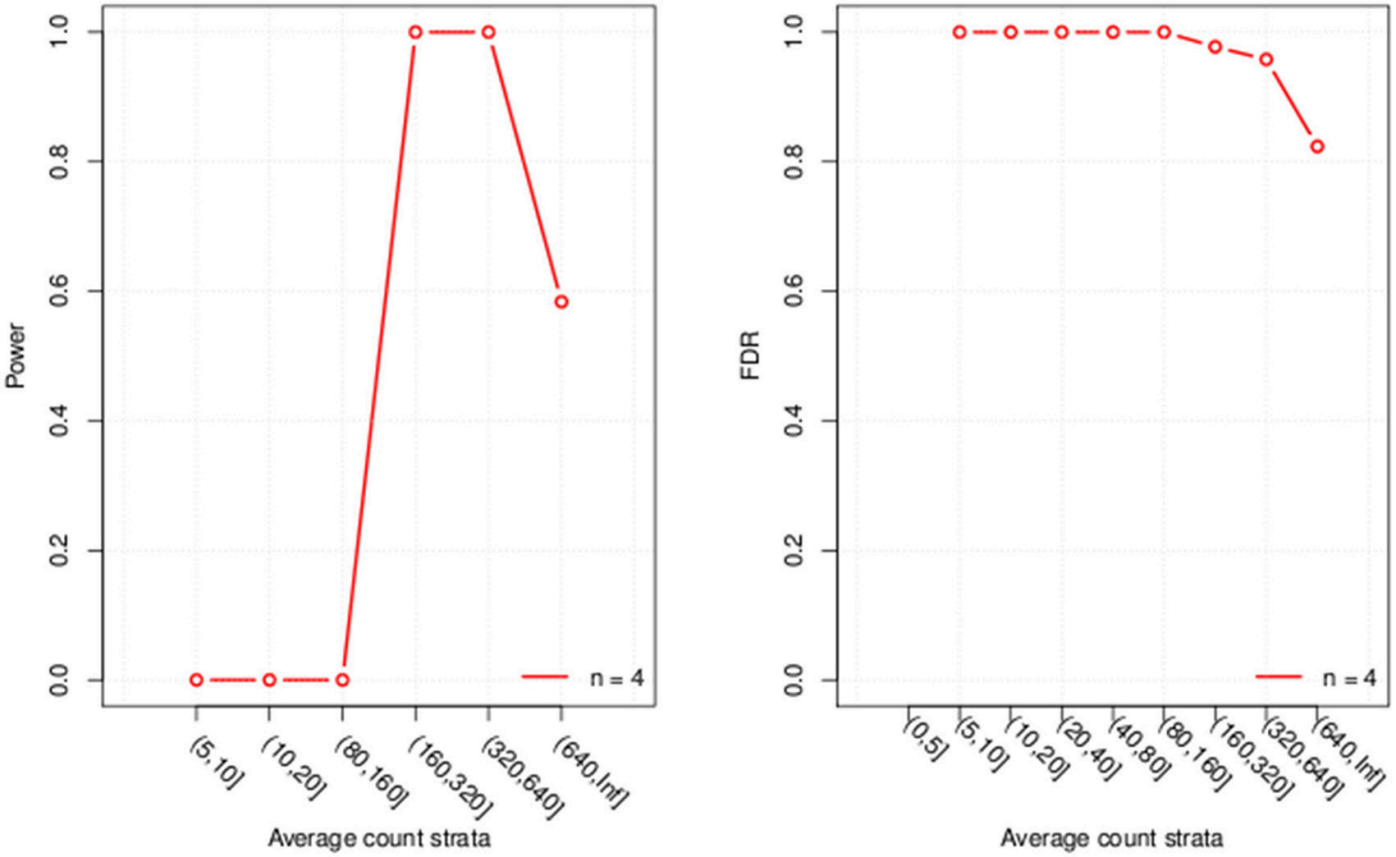
**Plots of the evaluated power and FDR for each of the six expression strata considered**.

Finally we visualize the results by generating a graph with 2 plots (Figure 1), one for power and one for FDR. If the parameter “error bar” is set to “TRUE” uncertainty bars are displayed around the estimates in the plot. For this demonstration we used a very small dataset which results in large uncertainty that might cause R to throw a warning message if the argument is set to “TRUE.”


  pdf("myPlots.pdf", width=10, height=7)
  par(mfcol=c(1,2), oma = c(0, 0, 2, 0))
  plotPower_ag(powers, error.bar=FALSE)
  plotFDR(powers, error.bar=FALSE)
  dev.off()


The code in this tutorial was run using R version 3.2.1, *PROPER* version 1.0.0, *EBSeq* version 1.10.0 and *edgeR* version 3.8.6.

## Author contributions

AG conducted the research and drafted the article.

### Conflict of interest statement

The author declares that the research was conducted in the absence of any commercial or financial relationships that could be construed as a potential conflict of interest.
